# The influence of NOx, temperature, wind and total radiation on the level of ozone concentration in the Upper Silesian agglomeration

**DOI:** 10.3389/fpubh.2024.1485333

**Published:** 2024-12-10

**Authors:** Joanna Kobza, Lechosław Dul, Mariusz Geremek

**Affiliations:** ^1^Department of Public Health, School of Public Health in Bytom Medical University of Silesia in Katowice, Piekarska, Poland; ^2^Department of Epidemiology and Biostatistics, School of Public Health in Bytom Medical University of Silesia in Katowice, Piekarska, Poland

**Keywords:** air pollution, ozone, environmental health, environmental policy, population health

## Abstract

In 2019, ozone was responsible for about 365,000 premature deaths worldwide (6.21 million healthy life years lost) and acute ozone exposure led to 16,800 premature deaths in the European Union. The aim of the study was to estimate the influence of NO, NO_2_, wind direction (WD) wind speed (WS), air temperature (TA), and total radiation (GLR) on ozone concentration levels. Data provided by 3 automatic air quality monitoring stations of the Regional Environmental Protection Inspectorate in Katowice, were used in this study. The measurements were conducted in from January 1 2009 to December 31 2017. The data obtained from the measuring stations were statistically analysed. The study showed that the strongest influencing factors for O_3_ values are air temperature and total radiation, with each showing a high correlation with ozone concentration. NO and NO_2_ had a dual effect on O_3_ concentration, causing an increase in ozone concentration at low NO and NO_2_ concentrations and a decrease in ozone concentration at higher NO and NO_2_ concentrations. We noted that the direction of the wind had very little effect on the concentration of O_3_. The influence of wind speed on the O_3_ level was also small, but stronger than that of the wind direction. The research shows that in the analysed years for selected measuring stations the strongest factors influencing O_3_ concentration are air temperature and total radiation, the NO and NO_2_ concentrations had a dualistic effect on the O_3_ concentration.

## Introduction

Indoor and outdoor air pollution is one of the leading threats to human life especially in low-and middle-income countries, therefore it is indicated as one of the main public health concerns (1–3). In 2019, air pollution led to 6.75 million early deaths (1 in 9 deaths globally) and 213 million years of healthy life lost (the 4th major mortality risk factor worldwide) (4).

Recent studies have confirmed that the high number of annual excess deaths are associated with high levels of ozone (O_3_), in 2019, for example, ozone accounted globally for about 365,000 early deaths (6.21 million years of healthy life lost) and acute ozone exposure led to 16,800 premature deaths in the European Union (5, 6). The European Environmental Agency report, ‘Air Quality in Europe 2022,’ indicated that ozone exposure above 70 μg/m^3^ led to 24,000 early deaths in the EU in 2020 (7). Ozone-related mortality could be decreased if stricter air quality standards are introduced (4).

Ozone (O_3_) consists of three oxygen atoms (an allotropic form of oxygen). Ninety-five percent of ozone is located in the stratosphere, at an altitude of 10–50 kilometres. Due to solar radiation, oxygen molecules (O_2_) are broken down into single atoms of oxygen (O). These single atoms react with other oxygen molecules to create ozone molecules. Due to the third atom of oxygen, ozone is a firm photochemical oxidant (8, 9). Stratospheric ozone significantly screens the lower atmospheric layers and the Earth’s surface from ultraviolet radiation (mostly UV-c and UV-b and half of the UV-a), but the tropospheric ozone, classified as one of the greenhouse gases, is proven to negatively influence human health (8, 9).

Tropospheric ozone (ground-level ozone) is synthesised by photochemical reactions between O_3_ precursors, such as nitrogen oxides (NOx), methane, carbon monoxide and volatile organic compounds (VOCs). Due to the importance of high temperatures and solar radiation, meteorological factors significantly influence the formation of ozone (9–11). Nowadays, the domination of anthropogenic emissions of ozone precursors into the atmosphere (for instance, due to industrial and construction activities, combustion processes, and vehicle traffic) is observed, thus a high concentration of tropospheric ozone is more likely in suburban areas than in city centres (10, 12, 13). The ozone precursor, methane (CH4), is the second most abundant greenhouse gas after carbon dioxide (CO_2_), and its atmospheric levels have increased significantly in recent years. Methane has natural and man-made sources (e.g., wetlands, peatlands and livestock) (14). Indirect sources of ozone are nitrogen oxides from forest fires, soil microbiological processes, and volatile organic compounds of vegetations (i.e., pinenes, terpenes), which increase the level of both NOx and VOC (15, 16). Meteorological conditions significantly affect ozone production at the regional and national levels (11, 12, 17). Long-range wind transportation of the tropospheric ozone and its precursors can occur (12, 14, 16, 18, 19).

### Aim

The aim of the study was to estimate the influence of NO, NO_2_, wind direction (WD), wind speed (WS), air temperature (TA), and total radiation (GLR) on ozone concentration levels.

## Methods

Data obtained from the Regional Environmental Protection Inspectorate in Katowice, capital of the Silesian province, and collected in the scope of National Environmental Monitoring were used in this study.

Data were provided by 3 automatic air quality monitoring stations of the Voivodeship Inspectorate for Environmental Protection, one station each in Katowice, Zloty Potok and Bielsko-Biała. The measurements were conducted in from January 12,009 to December 312,017.

The details of the station located in Złoty Potok, Częstochowa powiat, Janów commune are as follows: international code: PL0243A; measurement zone: Silesian agglomeration, urban background station; measurement target: plant protection; surroundings of the station: natural rural area, agricultural; location of the station: the area belonging to a Kamienna Góra forester’s lodge in Złoty Potok. The Janów commune is located approx. 20 km southeast of Częstochowa and approx. 25 km north of Zawiercie. The immediate surroundings of the station are meadows and plant-cultivated fields. At a distance of 150 m from the station, there is a forester’s lodge and several wood-fired summer cottages.

The details of the station located in Katowice (Kossutha St. 6, 40–844) are as follows: international code: PL0008A; measurement zone: Silesian agglomeration, urban background station; measurement target: human health protection; station surroundings: urban, residential; type of area: city (250–500 thousand inhabitants); surroundings of the station: to the north, blocks of flats, a railway line, a Motorway and the “Tysiąclecia” housing estate, to the east, commercial areas, to the south, residential buildings of the “Witosa” estate, and to the west, residential buildings and, further, post-mining areas of the Mine Hard Coal “Kleofas.”

The details of the station located in Bielsko-Biała, (Kossak-Szczuckiej St. 19, 43–300) are as follows: international code: PL0234A; measurement zone: Silesian agglomeration, urban background station; measurement target: human health protection; station surroundings: urban, residential; type of area: city (50–250 thousand inhabitants); location of the station: central part of Bielsko-Biała. The station is surrounded by high-and low-rise residential buildings. The surrounding buildings are heated from the heating network and individually.

The three monitoring stations were specifically selected for this research because of the different types of areas they are located in: one of the stations is located in a rural area, the other in a medium-sized city, and the station in Katowice is representative of the situation of a large city.

The data obtained from the measuring stations (NO, NO_2_, WD, WS, TA and GLR variables for ozone concentration O_3_) were statistically analysed using StatSoft, Statistica 13.1 software. A correlation analysis was performed. Pearson’s coefficient of linear correlation (R) and the partial correlation coefficient (R-part) were analysed. In addition to the multiple regression analysis (stepwise forward regression), the standardised regression coefficient *β* was calculated and analysed. When creating multiple regression models, the lagged variable O3 (d - 1) was used, whose values were the previous day’s O_3_ concentrations.

### Map

Location of the measurement stations (PL0234A PL0008A, PL0243A).

### Health effects

A study conducted in 21 East Asian cities between 1979 and 2010 showed significant associations between short-term ozone exposure and higher daily mortality rates. The results varied significantly from season to season, with a significant decrease recorded in winter at temperatures below 5°C, which is consistent with results obtained in Western European and North American countries (20).

Global warming significantly increases ground-level ozone and, in turn, the number of days with high concentrations of ozone (ozone seasons). As a highly reactive oxidant, ozone can interact with the cells of the cardiovascular and respiratory systems (21, 22).

The inflammation of the respiratory tract cells and the lungs is immune-dependent. Ozone exposure contributes to increased expression of mRNA of tumour necrosis factor-*α* (TNF-α), interleukin-1β (IL-lβ), interleukin-6 (IL-6) and interleukin-8 (IL-8) in human alveolar macrophages, and to increased concentrations of IL-6, IL-8 and fibrinogenic proteins in human airway epithelial cells (22).

Asthma is the commonest chronic respiratory disease in the world (23). In 2015, there were 9–23 million emergency visits globally due to the worsening of asthma symptoms (23). Exposure to ground-level ozone could increase acute hospital admissions of children and adolescents regardless of the geographical context (12). The examination of 3.959 children treated for acute asthma attack in the years 2016–2019 in Xiamen, China led to the conclusion that ozone concentration above 80 μg/m3 (O_3_-8 h) contributes to an increased risk of asthma attacks in children aged 0–14 (24). Short-term ozone exposures in urban areas (New York City, US) were significantly associated with asthma-related emergency department visits and hospitalisations in children aged 5–17 years (25). Human exposure to tropospheric ozone could intensify the severity of symptoms of respiratory tract infection like coughing or throat irritation (26).

Immune-dependent inflammation due to ozone exposure is also observed in the cardiovascular system (22). Increased levels of inflammatory cytokines, oxidative stress and immune-dependent inflammation of the endothelium could lead to a higher risk of cardiovascular diseases (CVD) due to ozone exposure (22, 27). Long-term exposure of 96,955 patients to ozone (89.7 μg/m^3^) was positively correlated with stroke, ischaemic heart disease and overall cardiovascular diseases (28). Long-term exposure to ozone was, however, also found to have inverse associations with CVD and respiratory mortality (29). The connection between short-term ozone exposure and cardio-respiratory mortality together with out-of-hospital cardiac arrest was evaluated for both first-time acute myocardial infarction patients and previously hospitalised patients (30, 31). Ozone exposure could be significantly associated with an increase in the number of years of life lost due to hypertension. A higher association was observed in elderly individuals born in autumn months. A statistically significant association was found for 0.68 year of life lost for every 10 μg/m^3^ increase in the ozone level (32).

A surface ozone level above 30 μg/m^3^ could cause epigenetic alterations and genotoxic effects, resulting in potentially severe health effects (33).

*In vitro* and *in vivo* tests confirmed that the ocular surface (i.e., its epithelial cells), which is directly exposed for external examination, could be easily damaged by ground-level ozone (34).

### Ozone regulations

According to the WHO, the daily concentration of O_3_ (daily maximum 8-h average) should not exceed 100 μg/m^3^ and a 0.3–0.5% growth in daily mortality has been observed for every 10 μg/m^3^ increase in 8-h ozone concentrations above 70 μg/m^3^ (35, 36).

It is important to note that the WHO guidelines are more restrictive than those implemented in the European Union.

The European Commission has recommended the acceptable level for O_3_ concentration as 120 μg/m^3^ (maximum daily 8-h mean), with permitted exceedances of up to 25 days per year (37).

The European Ozone Regulation No. 1005/2009 on ozone-depleting substances layer is a key piece of legislation in European Union (38). The act has two objectives: to implement the obligations of the Montreal Protocol on substances that deplete the O_3_ layer and to provide a higher level of protection in certain areas in the EU. The act is the basis for other European Commission regulations; Commission Regulation No. 537/2011 (39), Commission Regulation No. 291/2011 (40) and Commission Decision No. 2010/372/EU (41). The rules regarding the location and number of sampling points for air quality assessment, reference ozone measurement methods and data validation are regulated by Directive 2015/1480/EC of 28 August 2015 (37, 42). Issues of mutual exchange of information and reporting on air quality are regulated by the Commission Implementing Decision of 12 December 2011, which implemented Directives 2004/107/EC and 2008/50/EC of the European Parliament and Council (No. C/2011/9068) (43).

Based on the EU directives in Poland, the Regulation of the Ministry of Environment (44) clearly defines the terms assigned to individual ozone concentration levels by introducing the notion of target level, information level and alert level.

Target level is the level to which a substance in the air must decline within a certain period of time in order to reduce, avoid or prevent the harmful effects of the substance on human health and the environment. The target O_3_ level for a maximum daily average of 8 h is 120 μg/m^3^, with permissible exceedances for 25 days a year.

Information level is the level of a substance in the air above which there is a danger to human health deriving from short-term exposure. The information level for ozone-1-h average is 180 μg/m^3^.

Alert level is the level of a substance in the air above which there is a high risk to the health of the local population from short-term exposure to pollutants, and about which European Union States should take sudden action. The alarm level for O_3_-1-h average is 240 μg/m^3^.

If the O_3_ concentration in the air exceeds the alert level, it has a negative impact on human health. In such situations, vulnerable people, especially those affected by respiratory diseases, also children and the elderly, should avoid staying outside, while healthier people should reduce staying outside to the minimum.

## Results

The study showed that the strongest influencing factors for O_3_ values are air temperature (TA) and total radiation (GLR), with each showing a high correlation with ozone concentration (Tables 1–3). The regression coefficients (*β* coefficients) of TA and GLR in the multiple regression model were higher than those of the other variables.

**Table 1:** The bilateral linear relationship between NO, NO_2_, wind direction, wind speed, total radiation and air temperature on O3 values in Bielsko Biała measuring station PL0234A.

Measuring station	Year	Semester	R (O_3_ vs. Data)	R (O_3_ vs. NO)	R (O_3_ vs. NO_2_)	R (O_3_ vs. WD)	R (O_3_ vs. WS)	R (O_3_ vs. GLR)	R (O_3_ vs. TA)	R [O_3_ vs. O_3_(d-1)]
PL0234A	2009	I–VI	0.62	−0.62	−0.74	−0.13	0.25	MD	0.7	0.79
PL0234A	2009	VII–XII	−0.68	−0.63	−0.71	0.01	0.18	MD	0.78	0.79
PL0234A	2010	I–VI	0.55	−0.54	−0.72	−0.06	0.34	MD	0.68	0.77
PL0234A	2010	VII–XII	−0.63	−0.44	−0.6	−0.05	−0.03	MD	0.7	0.78
PL0234A	2011	I–VI	0.62	−0.56	−0.68	−0.17	0.1	MD	0.66	0.76
PL0234A	2011	VII–XII	−0.59	−0.56	−0.69	−0.13	0.12	MD	0.8	0.74
PL0234A	2012	I–VI	0.75	−0.68	−0.69	−0.17	−0.1	MD	0.83	0.79
PL0234A	2012	VII–XII	MD	−0.6	−0.63	−0.01	−0.08	MD	0.88	0.84
PL0234A	2013	I–VI	0.56	−0.63	−0.67	MD	MD	MD	MD	0.81
PL0234A	2013	VII–XII	−0.63	−0.57	−0.69	MD	MD	MD	MD	0.79
PL0234A	2014	I–VI	0.55	−0.6	−0.65	0.34	0.13	MD	0.76	0.7
PL0234A	2014	VII–XII	−0.54	−0.62	−0.75	0.38	0.3	MD	0.55	0.76
PL0234A	2015	I–VI	0.48	−0.61	−0.64	0.12	0.25	MD	0.67	0.69
PL0234A	2015	VII–XII	−0.72	−0.51	−0.64	−0.05	0.07	MD	0.86	0.85
PL0234A	2016	I–VI	0.66	−0.53	−0.74	−0.1	0.06	MD	0.76	0.72
PL0234A	2016	VII–XII	−0.56	−0.52	−0.69	0.14	0.14	MD	0.74	0.72
PL0234A	2017	I–VI	0.63	−0.63	−0.75	0.23	0.33	MD	0.73	0.76
PL0234A	2017	VII–XII	−0.62	−0.65	−0.7	0.01	0.13	MD	0.76	0.78

MD, missing data; author’s own study based on data of Regional Environmental Protection Inspectorate in Katowice.

**Table 2:** The bilateral linear relationship between NO, NO_2_, wind direction, wind speed, total radiation and air temperature on O3 values in Katowice measuring station PL0008A.

Measuring station	Year	Semester	R (O_3_ vs. Data)	R (O_3_ vs. NO)	R (O_3_ vs. NO_2_)	R (O_3_ vs. WD)	R (O_3_ vs. WS)	R (O_3_ vs. GLR)	R (O_3_ vs. TA)	R (O_3_ vs. O_3_ [d-1)]
PL0008A	2009	I–VI	0.65	−0.49	MD	−0.07	0.07	MD	0.66	0.81
PL0008A	2009	VII–XII	−0.73	−0.4	MD	0.05	−0.03	MD	0.77	0.82
PL0008A	2010	I–VI	0.64	−0.47	−0.62	0.19	0.15	0.69	0.64	0.7
PL0008A	2010	VII–XII	−0.76	−0.44	−0.51	0.11	−0.02	0.77	0.76	0.8
PL0008A	2011	I–VI	0.72	−0.43	−0.56	−0.07	0.02	0.69	0.62	0.76
PL0008A	2011	VII–XII	−0.68	−0.43	−0.44	−0.29	−0.2	0.81	0.81	0.8
PL0008A	2012	I–VI	0.78	−0.46	−0.61	−0.14	−0.06	0.78	0.78	0.8
PL0008A	2012	VII–XII	−0.84	−0.47	−0.46	0.13	−0.09	0.86	0.85	0.89
PL0008A	2013	I–VI	0.64	MD	MD	MD	MD	MD	MD	MD
PL0008A	2013	VII–XII	−0.65	−0.42	−0.36	−0.01	−0.06	0.73	0.78	0.79
PL0008A	2014	I–VI	0.7	−0.37	−0.45	0.16	−0.21	0.76	0.74	0.79
PL0008A	2014	VII–XII	−0.62	−0.44	−0.4	−0.02	0.28	0.75	0.55	0.71
PL0008A	2015	I–VI	0.69	−0.39	−0.51	0.31	0.36	0.78	−0.22	0.79
PL0008A	2015	VII–XII	−0.77	−0.41	−0.37	−0.19	−0.01	0.83	MD	0.84
PL0008A	2016	I–VI	0.72	−0.46	−0.5	−0.15	−0.01	0.78	0.74	0.75
PL0008A	2016	VII–XII	MD	−0.43	−0.31	0.13	−0.01	0.75	0.76	0.8
PL0008A	2017	I–VI	0.76	−0.51	−0.71	0.03	0.37	0.76	0.75	0.81
PL0008A	2017	VII–XII	−0.72	−0.45	−0.41	−0.12	0.08	0.79	0.8	0.81

MD, missing data; author’s own study based on data of Regional Environmental Protection Inspectorate in Katowice.

**Table 3:** The bilateral linear relationship between NO, NO_2_, wind direction, wind speed, total radiation and air temperature on O3 values in Złoty Potok measuring station PL0243A.

Measuring station	Year	Semester	R (O_3_ vs. Data)	R (O_3_ vs. NO)	R (O_3_ vs. NO_2_)	R (O_3_ vs. WD)	R (O_3_ vs. WS)	R (O_3_ vs. GLR)	R (O_3_ vs. TA)	R (O_3_ vs. O_3_ [d-1)]
PL0243A	2009	I–VI	0.62	−0.23	−0.43	0.01	0.02	MD	0.69	0.77
PL0243A	2009	VII–XII	−0.73	−0.45	−0.63	0.18	−0.07	MD	MD	0.8
PL0243A	2010	I–VI	0.28	−0.42	−0.36	0.001	0.25	MD	MD	0.67
PL0243A	2010	VII–XII	−0.69	−0.5	−0.57	0.09	0.04	MD	MD	0.78
PL0243A	2011	I–VI	0.62	−0.38	−0.63	−0.18	−0.26	MD	MD	0.67
PL0243A	2011	VII–XII	−0.67	−0.44	−0.72	−0.73	−0.26	MD	0.82	0.74
PL0243A	2012	I–VI	0.64	−0.34	−0.49	−0.24	0.48	MD	0.66	0.74
PL0243A	2012	VII–XII	−0.79	−0.57	−0.56	0.13	−0.08	MD	0.84	0.84
PL0243A	2013	I–VI	0.41	−0.48	−0.39	MD	MD	MD	MD	0.76
PL0243A	2013	VII–XII	−0.67	−0.45	−0.7	−0.37	0.24	0.26	0.75	0.7
PL0243A	2014	I–VI	0.53	−0.3	−0.5	0.1	0.03	0.74	0.58	0.74
PL0243A	2014	VII–XII	−0.65	−0.4	−0.54	0.2	0.14	0.74	0.64	0.78
PL0243A	2015	I–VI	0.6	−0.5	−0.5	0.1	−0.06	0.78	0.67	0.77
PL0243A	2015	VII–XII	−0.84	−0.4	−0.6	0.02	−0.25	0.82	0.88	0.89
PL0243A	2016	I–VI	0.69	−0.64	−0.7	0.06	−0.08	0.8	0.66	0.76
PL0243A	2016	VII–XII	−0.61	−0.49	−0.53	0.2	−0.04	0.72	0.76	0.8
PL0243A	2017	I–VI	0.6	−0.44	−0.56	0.14	0.28	0.69	0.62	0.68
PL0243A	2017	VII–XII	−0.65	−0.5	−0.6	0.06	−0.02	0.79	0.78	0.71

MD, missing data; author’s own study based on data of Regional Environmental Protection Inspectorate in Katowice.

**Figure 1:**
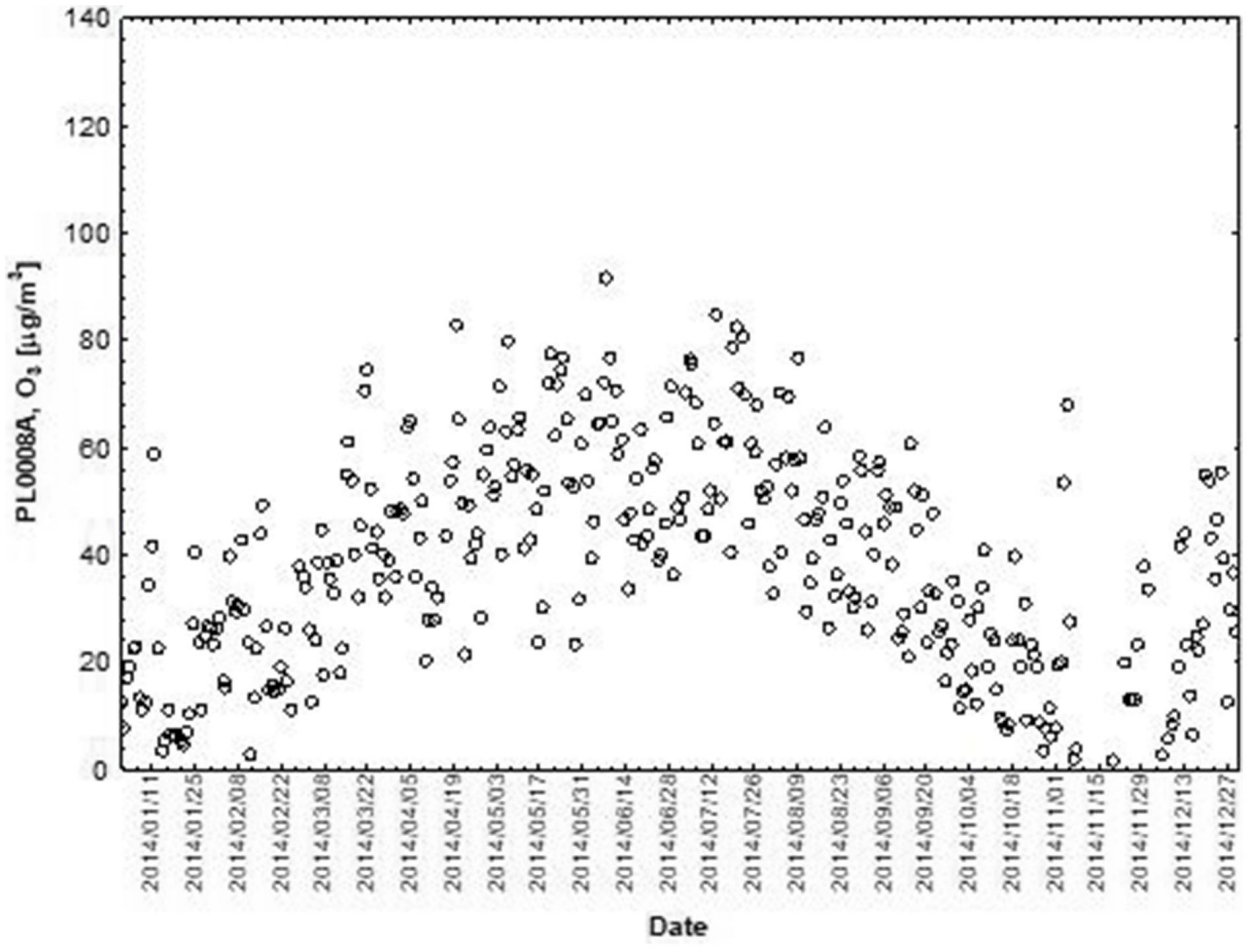
The concentration level of O_3_ (µg/m^3^) in 2014, PL0008A.

**Figure 2 fig2:**
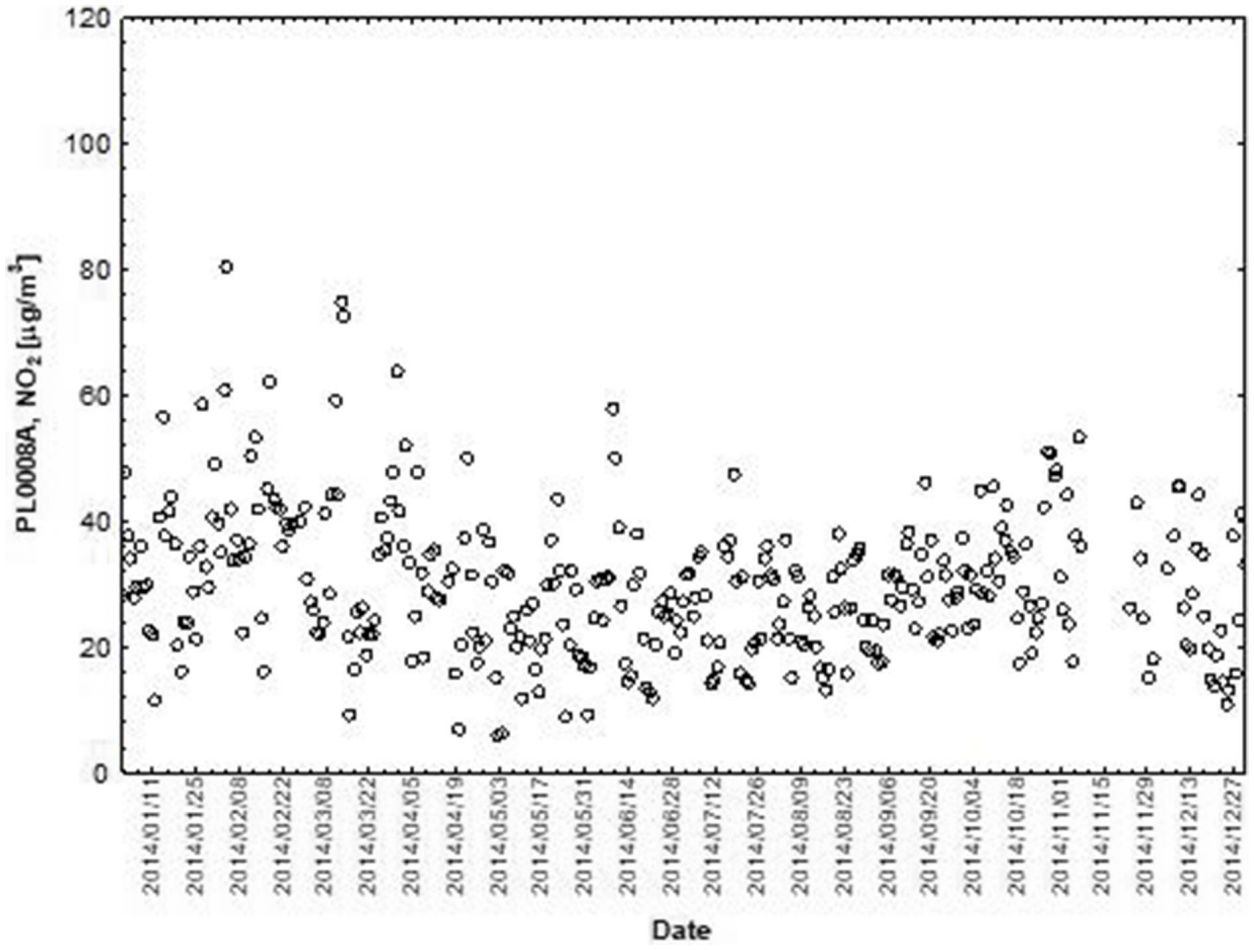
The concentration level of NO_2_ (µg/m^3^) in 2014, PL0008A, author’s own study based on data of Regional Environmental Protection Inspectorate in Katowice.

**Figure 3 fig3:**
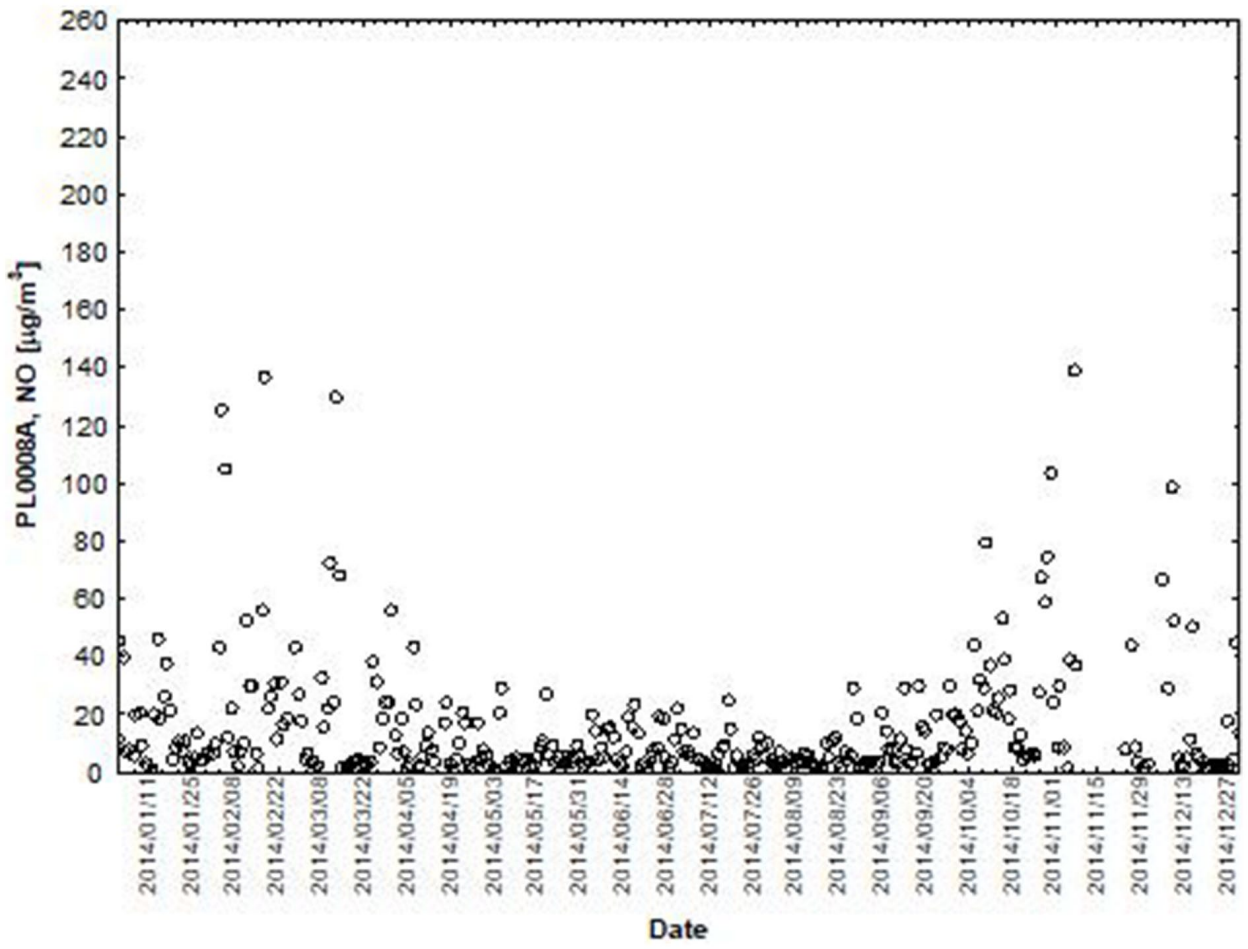
The concentration level of NO (µg/m^3^) in 2014, PL0008A author’s own study based on data of Regional Environmental Protection Inspectorate in Katowice.

**Figure 4 fig4:**
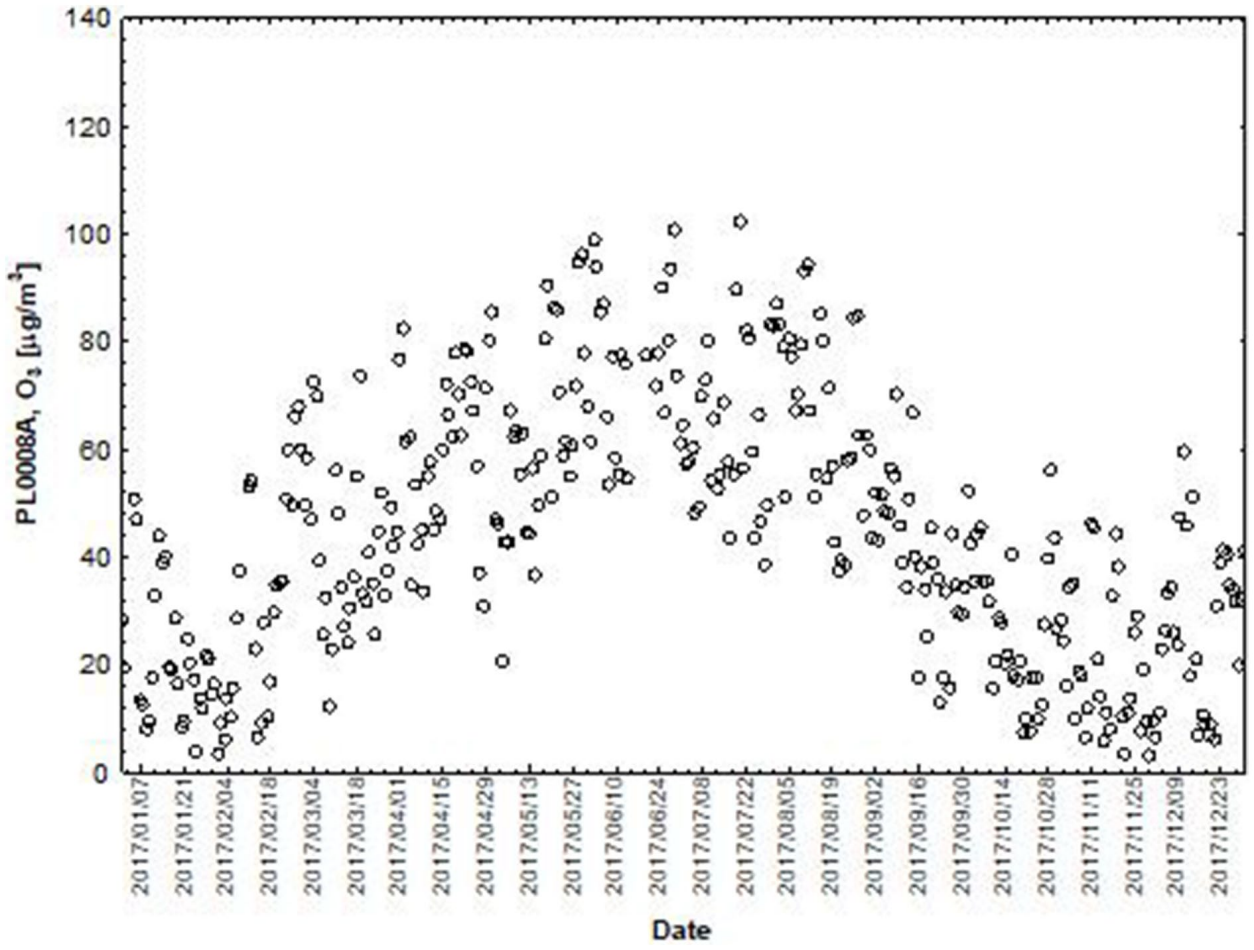
The concentration level of O_3_ (µg/m^3^) in 2017, PL0008A, author’s own study based on data of Regional Environmental Protection Inspectorate in Katowice.

**Figure 5 fig5:**
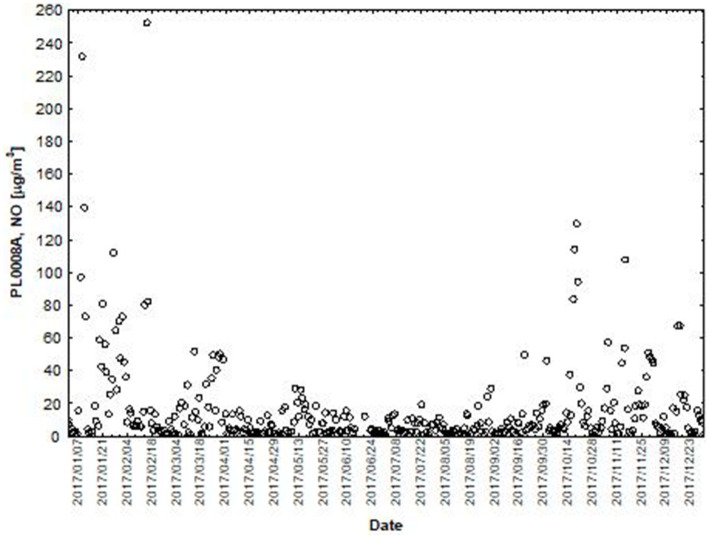
The concentration level of NO_2_ (µg/m^3^) in 2017, PL0008A, author’s own study based on data of Regional Environmental Protection Inspectorate in Katowice.

NO and NO_2_ had a dual effect on O_3_ concentration, causing an increase in ozone concentration at low NO and NO_2_ concentrations and a decrease in ozone concentration at higher NO and NO_2_ concentrations. From January to June, the level decreased and the O_3_ concentration increased in all selected measuring stations, however, from July to December, there was an increase in the level of NO_2_ and a decrease in the concentration of O_3_, a similar relationship occurred for NO and O_3_. As an example we present graphically the concentration level of O_3_, NO_2_ and NO in 2014 comparing to 2017 for the station located in Katowice (Figures 1–6). The relationships were confirmed by the values of the standardised regression coefficient *β* in the multiple regression model (Tables 4–6) and the R-part coefficient of partial correlation (Tables 7–9). We noted that the direction of the wind had very little effect on the concentration of O_3_. The influence of wind speed on O_3_ content was also small, but stronger than the wind direction. This can be seen from the values of the correlation coefficient R and the R-part (Tables 1–3, 7–9) for the statistical characteristics O_3_ and WS in comparison to O_3_ and WD. The values of the standardised regression coefficient β1 (Tables 4–6) confirm the above results.

**Figure 6:**
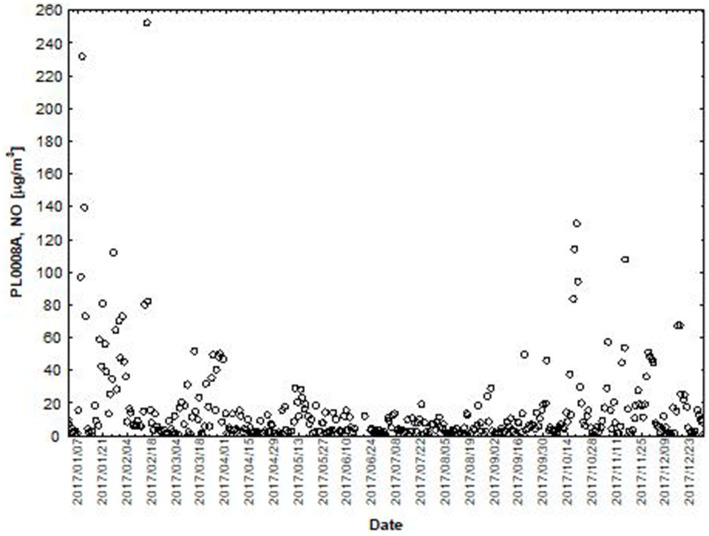
The concentration level of NO (µg/m^3^) in 2017, PL0008A, author’s own study based on data of Regional Environmental Protection Inspectorate in Katowice.

**Table 4:** The value of the standardised regression coefficient *β* of NO, NO_2_, wind direction, wind speed, total radiation and air temperature on O_3_ values in Bielsko Biała measuring station PL0234A.

Measuring station	Year	Semester	(NO)	(NO_2_)	(WD)	(WS)	(GLR)	(TA)	[O_3_(d-1)]
PL0234A	2009	I–VI	MD	−0.31	MD	0.1	MD	0.21	0.47
PL0234A	2009	VII–XII	−0.07	−0.2	−0.07	0.14	MD	0.35	0.42
PL0234A	2010	I–VI	MD	−0.21	−0.1	0.21	MD	0.27	0.46
PL0234A	2010	VII–XII	MD	−0.17	MD	0.07	MD	0.21	0.56
PL0234A	2011	I–VI	−0.16	−0.25	−0.13	MD	MD	0.14	0.44
PL0234A	2011	VII–XII	MD	−0.21	−0.13	0.15	MD	0.51	0.2
PL0234A	2012	I–VI	−0.25	MD	−0.14	MD	MD	0.41	0.33
PL0234A	2012	VII–XII	−0.09	MD	−0.11	0.17	MD	0.64	0.32
PL0234A	2013	I–VI	MD	MD	MD	MD	MD	MD	MD
PL0234A	2013	VII–XII	MD	MD	MD	MD	MD	MD	MD
PL0234A	2014	I–VI	−0.18	MD	MD	0.21	MD	0.54	0.28
PL0234A	2014	VII–XII	MD	−0.39	0.11	0.09	MD	0.13	0.45
PL0234A	2015	I–VI	−0.17	−0.06	MD	0.27	MD	0.43	0.33
PL0234A	2015	VII–XII	0.07	−0.25	MD	0.07	MD	0.44	0.41
PL0234A	2016	I–VI	0.07	−0.48	−0.07	MD	MD	0.22	0.37
PL0234A	2016	VII–XII	MD	−0.12	0.11	0.24	MD	0.49	0.38
PL0234A	2017	I–VI	MD	−0.25	MD	0.18	MD	0.36	0.32
PL0234A	2017	VII–XII	−0.11	−0.14	0.11	0.21	MD	0.47	0.33

MD, missing data; author’s own study based on data of Regional Environmental Protection Inspectorate in Katowice.

**Table 5:** The value of the standardised regression coefficient *β* of NO, NO_2_, wind direction, wind speed, total radiation and air temperature on O_3_ values in Katowice measuring station PL0008A.

Measuring station	Year	Semester	(NO)	(NO_2_)	(WD)	(WS)	(GLR)	(TA)	[O_3_(d-1)]
PL0008A	2009	I–VI	−0.14	MD	MD	0.18	0.52	MD	0.41
PL0008A	2009	VII–XII	−0.08	MD	0.04	0.17	0.49	0.1	0.41
PL0008A	2010	I–VI	MD	−0.28	0.06	0.21	0.48	MD	0.33
PL0008A	2010	VII–XII	MD	−0.19	0.07	0.12	0.5	MD	0.41
PL0008A	2011	I–VI	MD	−0.39	−0.06	0.04	0.43	MD	0.34
PL0008A	2011	VII–XII	0.09	−0.23	−0.16	0.28	0.48	0.26	0.3
PL0008A	2012	I–VI	MD	−0.3	−0.07	0.09	0.42	0.14	0.3
PL0008A	2012	VII–XII	−0.06	−0.05	MD	0.09	0.39	0.16	0.43
PL0008A	2013	I–VI	MD	MD	MD	MD	MD	MD	MD
PL0008A	2013	VII–XII	MD	−0.11	0.05	0.25	0.36	0.34	0.34
PL0008A	2014	I–VI	0.06	−0.26	MD	0.01	0.48	MD	0.45
PL0008A	2014	VII–XII	−0.06	−0.31	MD	MD	0.58	−0.06	0.35
PL0008A	2015	I–VI	−0.22	−0.21	0.09	0.3	0.16	MD	0.42
PL0008A	2015	VII–XII	MD	MD	MD	MD	MD	MD	MD
PL0008A	2016	I–VI	MD	−0.33	MD	0.09	0.58	MD	0.3
PL0008A	2016	VII–XII	MD	−0.11	0.07	0.24	0.4	0.25	0.38
PL0008A	2017	I–VI	0.24	−0.5	−0.05	0.13	0.44	MD	0.3
PL0008A	2017	VII–XII	MD	−0.21	−0.04	0.16	0.4	0.19	0.36

MD, missing data; author’s own study based on data of Regional Environmental Protection Inspectorate in Katowice.

**Table 6:** The value of the standardised regression coefficient *β* of NO, NO_2_, wind direction, wind speed, total radiation and air temperature on O_3_ values in Złoty Potok measuring station PL0243A.

Measuring station	Year	Semester	(NO)	(NO_2_)	(WD)	(WS)	(GLR)	(TA)	[O_3_(d-1)]
PL0243A	2009	I–VI	−0.17	−0.08	0.1	0.18	MD	0.43	0.46
PL0243A	2009	VII–XII	MD	MD	MD	MD	MD	MD	MD
PL0243A	2010	I–VI	MD	MD	MD	MD	MD	MD	MD
PL0243A	2010	VII–XII	MD	MD	MD	MD	MD	MD	MD
PL0243A	2011	I–VI	MD	MD	MD	MD	MD	MD	MD
PL0243A	2011	VII–XII	MD	MD	MD	MD	MD	MD	MD
PL0243A	2012	I–VI	−0.3	MD	−0.11	0.39	MD	0.57	0.23
PL0243A	2012	VII–XII	MD	MD	MD	0.07	MD	0.49	0.47
PL0243A	2013	I–VI	MD	MD	MD	MD	MD	MD	MD
PL0243A	2013	VII–XII	MD	MD	MD	MD	MD	MD	MD
PL0243A	2014	I–VI	−0.11	−0.15	MD	0.23	0.58	MD	0.4
PL0243A	2014	VII–XII	−0.13	MD	0.11	0.28	0.54	MD	0.33
PL0243A	2015	I–VI	−0.2	MD	0.05	0.22	0.51	MD	0.42
PL0243A	2015	VII–XII	−0.1	MD	MD	0.05	0.21	0.33	0.45
PL0243A	2016	I–VI	−0.21	−0.09	0.14	0.16	0.58	−0.08	0.3
PL0243A	2016	VII–XII	−0.05	−0.17	0.13	0.21	0.34	0.12	0.43
PL0243A	2017	I–VI	−0.23	MD	−0.04	0.24	0.58	−0.13	0.37
PL0243A	2017	VII–XII	−0.19	−0.09	0.07	0.18	0.46	0.17	0.24

MD, missing data; author’s own study based on data of Regional Environmental Protection Inspectorate in Katowice.

**Table 7:** The partial correlation of NO_2_, wind direction, wind speed, total radiation and air temperature and O_3_ values in Bielsko Biała measuring station PL0234A.

Measuring station	Year	Semester	R (O_3_ vs. Data)	R_part_ (NO)	R_part_ (NO_2_)	R_part_ (WD)	R_part_ (WS)	R_part_ (GLR)	R_part_ (TA)	R_part_ [O_3_(d-1)]
PL0234A	2009	I–VI	0.62	0.47	0.34	0.62	0.69	MD	0.01	0.24
PL0234A	2009	VII–XII	−0.68	−0.57	−0.52	−0.68	−0.77	MD	−0.06	−0.33
PL0234A	2010	I–VI	0.55	0.43	0.19	0.57	0.67	MD	−0.12	0.24
PL0234A	2010	VII–XII	−0.63	−0.53	−0.35	−0.63	−0.66	MD	0.02	−0.28
PL0234A	2011	I–VI	0.62	0.53	0.39	0.64	0.68	MD	0.18	0.27
PL0234A	2011	VII–XII	−0.59	−0.48	−0.42	−0.62	−0.65	MD	0.2	−0.29
PL0234A	2012	I–VI	0.75	0.68	0.69	0.75	0.8	MD	0.24	0.4
PL0234A	2012	VII–XII	MD	MD	MD	MD	MD	MD	MD	MD
PL0234A	2013	I–VI	0.56	0.39	0.31	MD	MD	MD	MD	0.22
PL0234A	2013	VII–XII	−0.63	−0.51	−0.49	MD	MD	MD	MD	−0.3
PL0234A	2014	I–VI	0.55	0.4	0.42	0.52	0.65	MD	−0.09	0.26
PL0234A	2014	VII–XII	−0.54	−0.37	−0.31	−0.51	−0.69	MD	0.02	−0.18
PL0234A	2015	I–VI	0.48	0.28	0.31	0.51	0.65	MD	−0.22	0.24
PL0234A	2015	VII–XII	−0.72	−0.67	−0.67	−0.72	−0.78	MD	0.01	−0.29
PL0234A	2016	I–VI	0.66	0.56	0.49	0.66	0.77	MD	0.03	0.36
PL0234A	2016	VII–XII	−0.56	−0.45	−0.32	−0.59	−0.69	MD	0.23	−0.24
PL0234A	2017	I–VI	0.63	0.46	0.37	0.62	0.78	MD	−0.04	0.3
PL0234A	2017	VII–XII	−0.62	−0.49	−0.5	−0.62	−0.75	MD	0.15	−0.27

MD, missing data; author’s own study based on data of Regional Environmental Protection Inspectorate in Katowice.

**Table 8:** The partial correlation of NO_2_, wind direction, wind speed, total radiation, air temperature and O_3_ values in Katowice measuring station PL0008A.

Measuring station	Year	Semester	R (O_3_ vs. Data)	R_part_ (NO)	R_part_ (NO_2_)	R_part_ (WD)	R_part_ (WS)	R_part_ (GLR)	R_part_ (TA)	R_part_ [O_3_(d-1)]
PL0008A	2009	I–VI	0.65	0.59	MD	0.67	0.71	0.2	0.23	0.26
PL0008A	2009	VII–XII	−0.73	−0.69	MD	−0.73	−0.76	−0.1	−0.18	−0.34
PL0008A	2010	I–VI	0.64	0.57	0.46	0.63	0.72	0.29	0.19	0.34
PL0008A	2010	VII–XII	−0.76	−0.72	−0.69	−0.76	−0.78	−0.35	−0.25	−0.38
PL0008A	2011	I–VI	0.72	0.69	0.67	0.72	0.74	0.39	0.46	0.38
PL0008A	2011	VII–XII	−0.68	−0.63	−0.63	−0.68	−0.73	−0.17	0.1	−0.28
PL0008A	2012	I–VI	0.78	0.77	0.75	0.78	0.82	0.43	0.38	0.41
PL0008A	2012	VII–XII	−0.84	−0.81	−0.81	−0.83	−0.85	−0.36	−0.34	−0.36
PL0008A	2013	I–VI	0.64	0.62	0.64	MD	MD	MD	MD	0.27
PL0008A	2013	VII–XII	−0.65	−0.63	−0.66	−0.65	−0.72	−0.07	0.07	−0.31
PL0008A	2014	I–VI	0.7	0.69	0.65	0.69	0.69	0.27	0.25	0.33
PL0008A	2014	VII–XII	−0.62	−0.57	−0.66	−0.55	−0.52	0.1	−0.19	−0.29
PL0008A	2015	I–VI	0.69	0.66	0.65	0.67	0.66	0.2	−0.22	0.32
PL0008A	2015	VII–XII	−0.77	−0.75	−0.76	−0.76	−0.8	−0.15	MD	−0.36
PL0008A	2016	I–VI	0.72	0.69	0.7	0.72	0.8	0.23	0.22	0.38
PL0008A	2016	VII–XII	MD	MD	MD	MD	MD	MD	MD	MD
PL0008A	2017	I–VI	0.76	0.71	0.66	0.76	0.81	0.35	0.33	0.39
PL0008A	2017	VII–XII	−0.72	−0.69	−0.72	−0.71	−0.79	−0.17	−0.02	−0.32

MD, missing data; author’s own study based on data of Regional Environmental Protection Inspectorate in Katowice.

**Table 9:** The partial correlation of NO_2_, wind direction, wind speed, total radiation, air temperature and O_3_ values in Złoty Potok measuring station PL0243A.

Measuring station	Year	Semester	R (O_3_ vs. Data)	R_part_ (NO)	R_part_ (NO_2_)	R_part_ (WD)	R_part_ (WS)	R_part_ (GLR)	R_part_ (TA)	R_part_ [O_3_(d-1)]
PL0243A	2009	I–VI	0.62	0.7	0.52	0.63	0.64	MD	−0.09	0.26
PL0243A	2009	VII–XII	−0.73	−0.71	−0.55	−0.72	−0.75	MD	MD	−0.35
PL0243A	2010	I–VI	0.28	0.07	0.02	0.28	0.36	MD	MD	0.11
PL0243A	2010	VII–XII	−0.69	−0.55	−0.4	−0.69	−0.72	MD	MD	−0.33
PL0243A	2011	I–VI	0.62	0.6	0.33	0.61	0.57	MD	MD	0.23
PL0243A	2011	VII–XII	−0.67	−0.69	−0.5	MD	MD	MD	0.11	−0.33
PL0243A	2012	I–VI	0.64	0.64	0.55	0.2	0.41	MD	0.26	0.34
PL0243A	2012	VII–XII	−0.79	−0.7	−0.69	−0.79	−0.81	MD	−0.19	−0.37
PL0243A	2013	I–VI	0.41	0.54	0.47	MD	MD	MD	MD	0.18
PL0243A	2013	VII–XII	−0.67	−0.58	−0.42	MD	MD	MD	MD	−0.3
PL0243A	2014	I–VI	0.53	0.5	0.26	0.54	0.57	0.04	0.15	0.23
PL0243A	2014	VII–XII	−0.65	−0.68	−0.49	−0.64	−0.76	−0.07	−0.2	−0.3
PL0243A	2015	I–VI	0.6	0.49	0.46	0.6	0.65	0.07	0.08	0.27
PL0243A	2015	VII–XII	−0.84	−0.82	−0.74	−0.84	−0.84	−0.42	−0.4	−0.37
PL0243A	2016	I–VI	0.69	0.52	0.36	0.69	0.71	0.14	0.31	0.35
PL0243A	2016	VII–XII	−0.61	−0.51	−0.42	−0.62	−0.63	−0.08	0.21	−0.25
PL0243A	2017	I–VI	0.6	0.53	0.4	0.59	0.66	0.13	0.16	0.32
PL0243A	2017	VII–XII	−0.65	−0.62	−0.47	−0.65	−0.7	−0.04	0.12	−0.34

MD, missing data; author’s own study based on data of Regional Environmental Protection Inspectorate in Katowice.

## Discussion

The concentration of ozone near the earth’s surface depends on the processes of ozone formation and dispersion. The process of ozone formation depends mainly on ozone precursors, while ozone dispersion depends on meteorological conditions. In recent years, tropospheric ozone levels in many parts of the world have shown significant regional and global responses to meteorological (climatic) changes (45). Our research shows that temperature and radiation are important factors that strongly affect the level of O_3_ concentration. Research conducted in Nicosia, Cyprus form 2007 to 2014 provided similar results. It examined how ozone levels changed during a heat wave (defined as 4 consecutive days with a daily maximum temperature above 39°C) compared to summer conditions without a heat wave. There was a medium to strong positive correlation between ozone and temperature, as well as between ozone and daytime UVA and UVB radiation, which increased by about 35% in heat wave conditions (46). The results of another study, using data from 17 measurement stations in Sydney, Australia, showed that during periods of extreme heat, the impact of temperature on air quality was as large as the impact of biogenic emissions (eucalyptus trees are key to biogenic emissions in Australia) (47). Studies carried out in Lithuania showed that the concentration of ozone increased with increasing temperature; however, ozone concentration was not significantly related to relative air humidity, and a decrease in ozone concentration was observed during the rainy season (48). Similarly, in a study conducted in the Ciuc Basin, Romania, the intensity of sunlight and increase in temperature had a significant impact on the increase in ozone pollution (49). The relationship between the maximum daily temperature and changes in ground-level ozone concentrations was investigated in the state of Terengganu, Malaysia, using data from 2000 to 2010 (excluding 2008); measurements made at two stations representing urban and industrial areas were analysed. The study found a positive linear correlation between the maximum daily temperature and the maximum daily ozone concentration, with levels higher in the industrial area than in the urban area (50). The Terengganu results also confirmed that temperature played a key role in shaping ground-level ozone concentrations. In addition, ozone concentrations were highest in dry and warm air masses during the southwest monsoon and were usually associated with haze episodes in the Malaysian peninsula (50). Similarly, the direct relationship between temperature and ozone was confirmed by studies carried out in Porto (51).

However, not all studies provide such unambiguous results. For example, studies conducted in eastern Texas showed that the temperature in this area rarely had any effect on ozone concentrations, perhaps due to the small spatial variation (52).

Measurements of the solar ultraviolet index (UVI) in Arica, Chile from 2006 to 2015 indicated that 16.6% of the maximum daily UVI measured at solar noon during the summer season was high and very high (8 < UVI < 10) while 83.1% of the measured maximum daily UVI was extreme (> 11 UVI), according to the WHO scale (53). Hourly and daily changes in ground-level ozone were also analysed in relation to meteorological parameters (UVB radiation) in the Baltic Sea region in Lithuania. A close correlation was established between ground-level ozone concentration and UVB radiation intensity (48).

In our research, we also tried to estimate the influence of wind strength and direction on ozone concentration. The influence of wind speed on the O_3_ level was small but stronger than the influence of wind direction. A similar analysis in Cyprus showed a slight decrease in wind speed during heat waves, leading to stagnant weather conditions. The same study analysed a diurnal cycle of wind speeds that peaked at noon when ozone levels were highest (46). In studies conducted in Porto, the highest concentrations of O_3_ were observed at high wind speeds (51). On the other hand, studies carried out in Lithuania showed that the dominance of wind direction had a significant impact on the variability of ozone concentrations. An unusual situation was observed in terms of wind dominance, namely the wind from the continent was twice as frequent as the wind from the sea. Meteorological parameters such as relative humidity, wind speed and direction had different effects on the ozone concentration at the ground level of the atmosphere. Wind direction had the greatest impact on the variability of ozone concentration. Higher concentrations of ozone in the boundary layer of the atmosphere were found when the wind was blowing from the Baltic towards the continent, perhaps due to the low rate of ozone decomposition (48). Modelling and statistical analyses described three important variables that could affect O_3_ concentration, *viz.*: wind speed, temperature and NO_2_ concentration (51).

Our research showed that from January to June in all the measurement stations, the level of NO_2_ and NO decreased while the concentration of O_3_ increased. In contrast, we observed an increase in NO_2_ and NO levels but a decrease in O_3_ concentrations from July to December. Similar results were obtained in a study conducted in 2005–2010 for four different types of stations in central Poland. On the basis of daily averages, it was found that the seasonal maximum of ozone occurred in spring and early summer, and the minimum in autumn (54). In addition, the same study showed that the lowest ozone concentration occurred in the early morning and the maximum in the afternoon. Moreover, ozone concentrations differed significantly on weekends and weekdays. The measurement results also showed that the concentration of surface ozone was higher in rural areas than in urban or suburban areas (54).

In the above-mentioned study conducted in Cyprus during the heat wave period, a negative correlation of NOx concentration with ozone levels was observed, increasing during the heat wave conditions, leading to steeper ozone minima in the morning (46). Also, the research carried out in Porto (Jan 2012–Dec 2013) confirmed unequivocally that the highest O_3_ concentrations were observed for low NOx concentrations (51).

The results of the East Texas study showed that the spatial mean of ground-level ozone concentrations was strongly related to the spatial mean of NO_2_ concentrations, although the spatial distributions of NO_2_ and ozone concentrations were not uniform throughout the study period due to uneven wind speeds and directions. Thus, this study confirmed that wind speed and direction also played a significant role in ozone spread (52). Interesting conclusions were provided by the results of research on the sources of surface ozone in Switzerland in the summer (June–August) of 2018, one of the warmest years in Europe. The largest share in the formation of surface ozone was border import (65%), followed by off-road traffic (11%) and road traffic (8%). Compared to 2000, lower emissions were recorded in 2018, leading to a reduction in ozone levels in most areas of the country with the exception of some urban areas, with the largest decrease being in emissions due to road traffic. The results indicated that reduced anthropogenic emissions were the main reason for the reduction of high ozone concentrations during the hot summer in Switzerland (55). The so-called border import is quite important in many countries, especially transit countries. In Romania, for instance, the air masses flowing through the Ciuc Basin in 2008–2017 came from neighbouring Central and Eastern European countries. Local sources of NOx emissions in this region are car exhaust fumes emitted in the form of NO and burning biomass in cold winter periods (49).

Currently, ozone probes, satellites and commercial aircraft provide information on the distribution of tropospheric ozone. Long-term surface observations have limited global spatial coverage, but data from various locations indicate that ozone concentrations in the 21st century are higher than in the 1970s and 1980s, and we have seen an increase in tropospheric ozone concentrations since the 1990s. Some highly polluted regions of East Asia have seen increases in ozone concentrations since 2000, while many other regions have seen decreases, so there is no clear global trend in surface ozone concentrations since 2000. Satellite data can estimate the global effect of long-wave ozone radiation, but this assessment is difficult due to the limited number of observations in places where the radiation effect is greatest (56). Extensive air quality monitoring and analysis has shown that reductions in ozone precursor emissions have reduced extreme levels of ozone across much of Europe, both in rural and urban areas, as well as in North America (57). Between 2000 and 2017, rural areas across Europe showed an overall decline in ozone concentrations while highly urbanised areas showed an increase in ozone (58, 59).

Recent modelling projections predict that future climate change will increase surface ozone levels in polluted regions and decrease global ozone levels due to stronger chemical ozone loss (45). However, uncertainties in climate–ozone responses and limitations in the capabilities of model analyses still pose challenges to such predictions. Experts highlight the growing importance of future increases in stratosphere–troposphere exchange in modulating tropospheric ozone, which can largely offset the predicted chemical loss of tropospheric ozone load. They also highlight that uncertainties in isoprene chemistry, biogenic emissions associated with changing CO_2_ levels and vegetation, and interactions between ozone and vegetation can greatly influence surface ozone levels and, consequently, climate change (45). Our results will contribute to more targeted environmental policies and improve the health risk assessment of the effects of ozone pollution on local populations.

## Conclusion

The research shows that in the analysed years for selected measuring stations, the NO and NO_2_ concentrations had a dualistic effect on the O_3_ concentration, leading to an increase in the O_3_ concentration level at low concentrations and a decrease in the O_3_ level at higher concentrations. The strongest factors influencing O_3_ concentration are air temperature and total radiation. The local policy makers responsible for environmental policy should take it into consideration.

### Limitations of the study

It should be emphasised that analysing the correlation between statistical characteristics is not tantamount to strictly comparing their physical and chemical properties and thus may lead to false conclusions, including the finding of illusory correlation, which indicates a relationship between variables when no relationship exists. The observed statistical relationships do not necessarily imply true cause-and-effect relationships between the components of the atmosphere.

### Recommendations

Further studies are needed to explain the dual effect of NOx on O3 concentration and its dependence on atmospheric conditions.

## Data Availability

The raw data supporting the conclusions of this article will be made available by the authors, without undue reservation.
